# Supplementary material for: A Distinct Hibiscus sabdariffa Extract Prevents Iron Neurotoxicity, a Driver of Multiple Sclerosis Pathology
Source: Cells. 2022 Jan 27;11(3):440. doi: 10.3390/cells11030440 (PMC8834068; doi:10.3390/cells11030440)
Supplement: Supplementary file 1 [file cells-11-00440-s001.zip › cells-1536422-supplementary.pptx]

## Slide 1
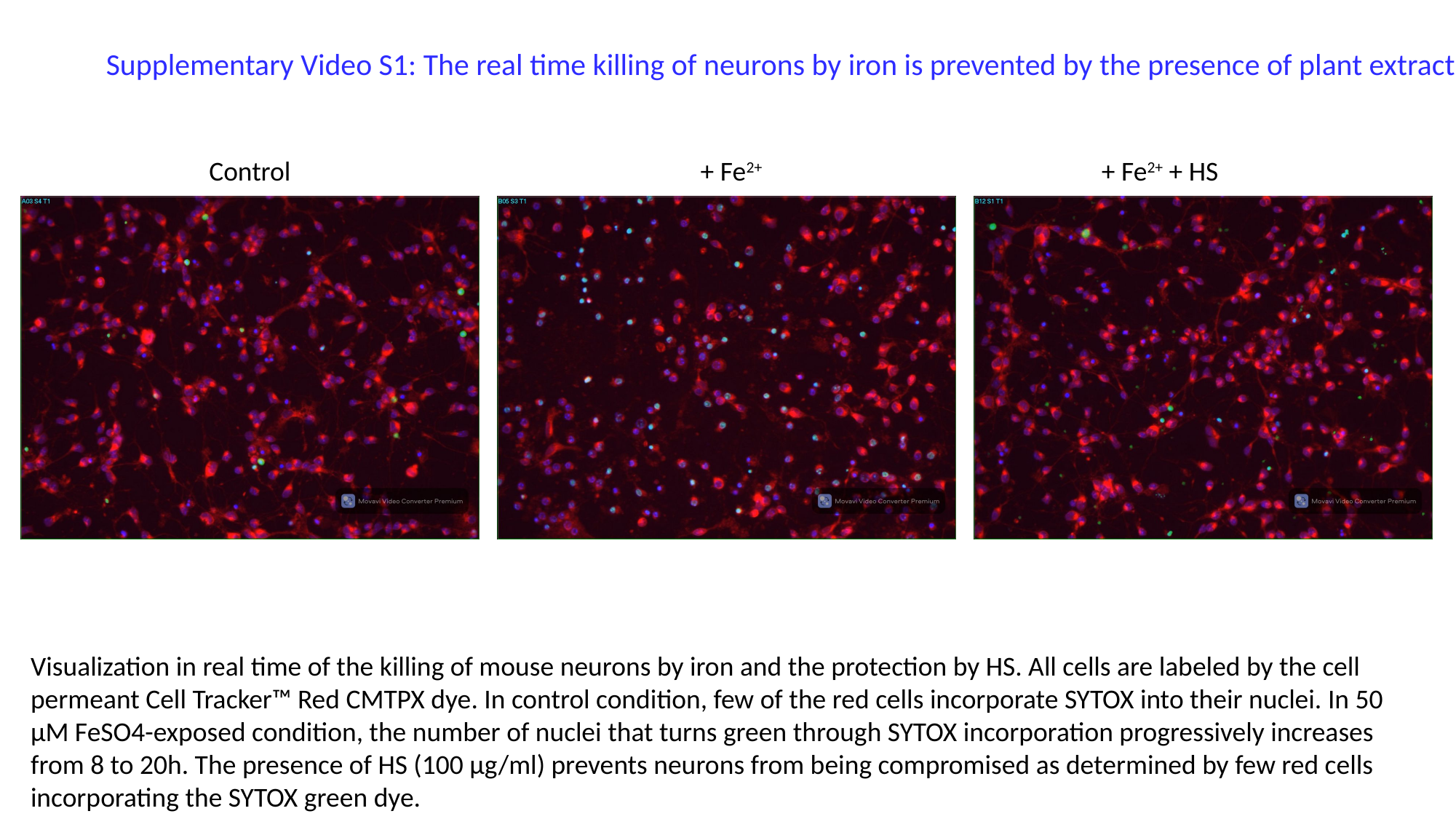

Supplementary Video S1: The real time killing of neurons by iron is prevented by the presence of plant extract
Control
+ Fe2+
+ Fe2+ + HS
Visualization in real time of the killing of mouse neurons by iron and the protection by HS. All cells are labeled by the cell permeant Cell Tracker™ Red CMTPX dye. In control condition, few of the red cells incorporate SYTOX into their nuclei. In 50 µM FeSO4-exposed condition, the number of nuclei that turns green through SYTOX incorporation progressively increases from 8 to 20h. The presence of HS (100 µg/ml) prevents neurons from being compromised as determined by few red cells incorporating the SYTOX green dye.

## Slide 2
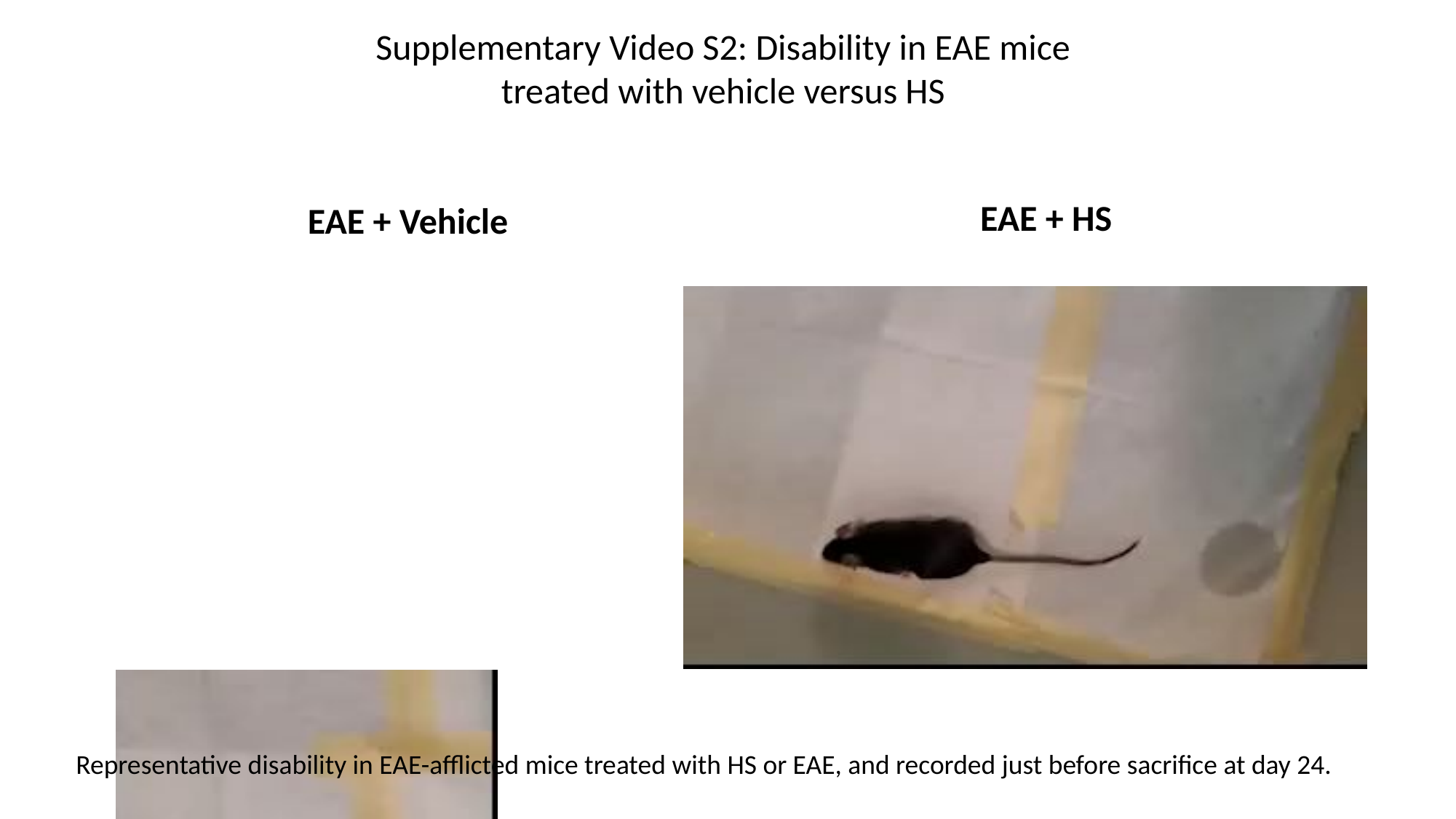

Supplementary Video S2: Disability in EAE mice
treated with vehicle versus HS
EAE + HS
EAE + Vehicle
Representative disability in EAE-afflicted mice treated with HS or EAE, and recorded just before sacrifice at day 24.
